# A Novel Phytomyxean Parasite Associated with Galls on the Bull-Kelp *Durvillaea antarctica* (Chamisso) Hariot

**DOI:** 10.1371/journal.pone.0045358

**Published:** 2012-09-17

**Authors:** Franz Goecke, Jutta Wiese, Alejandra Núñez, Antje Labes, Johannes F. Imhoff, Sigrid Neuhauser

**Affiliations:** 1 Kieler Wirkstoff-Zentrum, Helmholtz Centre for Ocean Research GEOMAR, Kiel, Schleswig-Holstein, Germany; 2 Facultad de Ecología y Recursos Naturales, Universidad Nacional Andrés Bello, Santiago de Chile, Región Metropolitana, Chile; 3 Institute of Microbiology, University of Innsbruck, Innsbruck, Tyrol, Austria; University of Melbourne, Australia

## Abstract

*Durvillaea antarctica* (Fucales, Phaeophyceae) is a large kelp of high ecological and economic significance in the Southern Hemisphere. In natural beds along the central coast of Chile (Pacific Ocean), abnormal growth characterized by evident gall development and discolorations of the fronds/thallus was observed. Analysing these galls by light microscopy and scanning electron microscopy revealed the presence of endophytic eukaryotes showing typical characteristics for phytomyxean parasites. The parasite developed within enlarged cells of the subcortical tissue of the host. Multinucleate plasmodia developed into many, single resting spores. The affiliation of this parasite to the Phytomyxea (Rhizaria) was supported by 18S rDNA data, placing it within the Phagomyxida. Similar microorganisms were already reported once 23 years ago, indicating that these parasites are persistent and widespread in *D. antarctica* beds for long times. The symptoms caused by this parasite are discussed along with the ecological and economic consequences. Phytomyxean parasites may play an important role in the marine ecosystem, but they remain understudied in this environment. Our results demonstrate for the first time the presence of resting spores in Phagomyxida, an order in which resting spores were thought to be absent making this the first record of a phagomyxean parasite with a complete life cycle so far, challenging the existing taxonomic concepts within the Phytomyxea. The importance of the here described resting spores for the survival and ecology of the phagomyxid parasite will be discussed together with the impact this parasite may have on ‘the strongest seaweed of the world’, which is an important habitat forming and economic resource from the Southern Hemisphere.

## Introduction

*Durvillaea antarctica* (Chamisso) Hariot is a large brown seaweed which belongs to the order Fucales (Phaeophyceae) [1]. It has a subantarctic distribution limited to the Southern Hemisphere, specifically South America, New Zealand and subantarctic islands [2], [3]. This algal species plays an important role in the occupation of habitat and the structuring of coastal communities. It frequently dominates the intertidal and shallow subtidal flora in regions with a stable, rocky substratum that is exposed to wave force [4]. *D. antarctica* is the dominant primary producer and the primary repository of organic material and energy in these environments [5]. Like other large brown algae in temperate coasts of the world, *Durvillaea* spp. modifies the microenvironments by providing much of the vertical structure which is then inhabited by creatures belonging to all domains of life [6], [7]. Its long, floating fronds can reach a length of more than 15 m [8], and its holdfast by itself constitutes a temporal or permanent habitat for a rather large number of different species of invertebrates by giving them shelter, by conforming spawning and habitat substrates, and by minimizing wave and predation pressure [9].

Kelp species (common name for large brown seaweeds) have also local economic and social importance since many local inhabitants base their living on the resources provided directly or indirectly by these kelps. In Chile the stipe and dried fronds of *D. antarctica*, locally known as “*cochayuyo*”, have been harvested for human consumption already by the *Mapuche* culture (prior to pre-Spanish settlement), a tradition that is now continued by modern intertidal subsistence food-gatherers and artisanal fishers who also sell it in local markets [10]. Bull-kelp is considered as a good source of fibres and polysaccharides including hydrocolloids such as alginic acid [11], [12]. Consequently, *D. antarctica* has been heavily exploited and exported as raw material for the extraction of those alginates which have wide applications in food and pharmaceutical industries [13], [14]. Chile produces 10% of the global brown seaweed supply [15], with landings of *ca*. 2600 (±900) wet metric tons (Mg) *D*. *antarctica* per year [10]. This global demand of algal products in cosmetic, food, and pharmaceutical industries has increased and expanded considerably over the last decades, increasing the need for the development of strategies to lower the frequency and abundance of infections with pathogens but also of strategies improving the protection of these algae [16]. Algal diseases are more under the spotlight than ever before [17]. First, there is considerable concern that climate change and other anthropogenic stressors may increase the spread of pathogens, enhance their virulence as well as decrease the resilience of marine host organisms like algae [18]. Second, an alarming decrease in the density and biomass of canopy-forming kelps has been reported worldwide [19].

Infections with microbial pathogens cause a variety of physiological reactions of the host, and those infections may cause obvious changes in macroalgal morphology, like galls, appearance of holes, discolorations, but sometimes infections do not produce any visual changes at all [20]. Galls and tumour-like structures can be found on numerous macroalgae. A variety of viruses, bacteria, fungi, microalgae, nematodes and copepods can be associated with disease symptoms as can be industrial pollutants or abiotic factors [21], [22]. Only few notorious parasites or obligate epiphytes are so far known to be associated with the genus *Durvillaea* [23], [24]. The most obvious, *Herpodiscus durvillaeae* (Lindauer) South is an obligate parasitic brown alga restricted to New Zealand [25], which produces velvety red-brown patches on the host frond surface [26]. *D. antarctica* has developed special adaptations (e.g. thallus morphology, high alginate content, stark holdfast) to withstand extreme high hydrodynamic environment [11]. Thus, modifications of the natural thallus characteristics (by galls or holes) interact with these vital features and consequently can affect the survival of the host [27].

**Figure 1:**
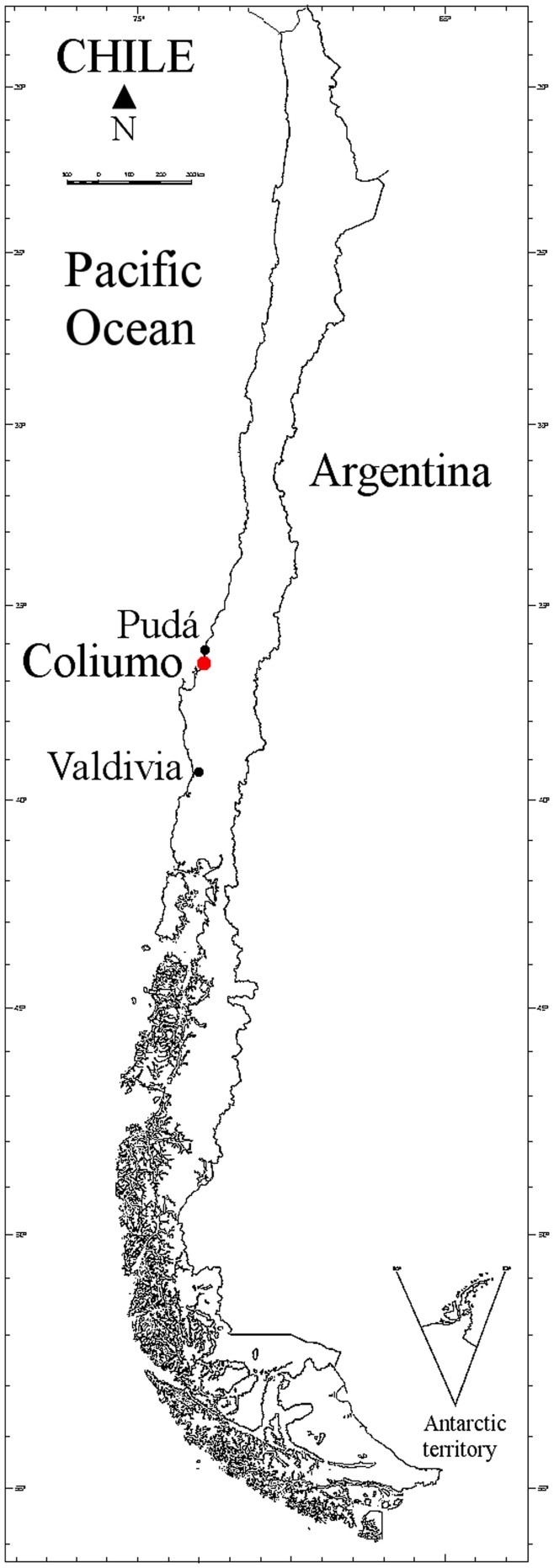
Locations where galls of *Durvillaea antarctica* caused by phytomyxean parasites were found in this study or by Aguilera et al. [27]. In 2011 the parasite was found in Necochea beach at Coliumo bay (in red), at the central coast of Chile, Pacific Ocean (map from [36], modified). Similar symptoms were reported from *D. antarctica* at the localities of Pudá and Valdivia [27], in black).

In Chile, twenty three years ago, in the area of Valdivia and Concepción (Pudá beach) (Fig. 1), the presence of an endophytic parasite associated with galls in *D. antarctica* was reported by Aguilera et al. [27]. Based on morphological characteristics, the authors thought that the parasite belongs to the Phytomyxea (then referred to as Plasmodiophorales). Their observations were based on light microscopy only, and the ultrastructure and/or development of the parasite were not studied in detail. This lack of descriptions of special morphological characteristics such as form and distribution of resting spores and the observation of the characteristic cruciform nuclear division, was the reason why Maier et al. [28] questioned the placement of this parasite in the Phytomyxea. Since this time there have to our knowledge not been any further records of this parasite in the scientific literature.

Here we describe an epidemic of unusual galls formed on the fronds of *D. antarctica* occurring at the central coast of Chile in summer 2011. On infected plants, lesions, galls and discolorations were observed on the fronds of this important algal resource. These lesions were analysed using molecular and microscopical techniques. The symptoms of the disease will be described on the macroscopic and microscopic scale. We also confirm the affiliation of this parasite to the Phytomyxea based on molecular data. The local and national economic importance of *D. antarctica* together with its ecological role for other organisms (*i.e.* fish and crustacean), some of them being economical resources themselves add importance to a better knowledge about the parasites affecting the health of *D. antarctica*.

## Materials and Methods

### Field Sampling

Fresh samples of *D*. *antarctica* (n = 2) were collected stranded after a night storm from Necochea beach, Coliumo bay (36°31’36’’S; 72°57’24’’W), central coast of Chile, Pacific Ocean (Fig. 1). No specific permits were required for the described field studies.

Pieces of different sections of diseased and healthy thalli were collected from the intertidal zone in summer (January) 2011; were carefully cut with a knife and transferred into sterile plastic bags. In the laboratory, the macroalgae were rinsed three times with sterile seawater to remove associated debris, planktonic and loosely attached microorganisms. Sections of 1 cm^2^ of the thallus were prepared by fixation with 2% formaldehyde in seawater for microscopy, and in 95% ethanol for the molecular analysis.

### Light Microscopy and Characterization of the Microorganisms

To observe cellular anomalies, pigmentation or damage in algal tissue small pieces of fresh material (1 cm^2^) of the thallus were cut with a razor blade (sections of approx. 40 µm), placed on slides and stained with a mixture of 1.0% aniline blue with 30% Karo Syrup acidified with HCl or similarly mounted but not stained [29]. For the photomicrographs, we used a Olympus (CX21) microscope equipped with a camera. The images were compared to specialized literature on morphology, reproduction and cytology of *D*. *antarctica i.e.* [30]–[34].

Samples were cut into thin sections using a sterile razor blade and mounted in Vectashield with DAPI. The samples where then carefully squashed and analysed using a Nikon Optiphot light microscope (Nomarski interference contrast and epifluorescence 330–380 nm, oil immersion objective 100x).

### Scanning Electron Microscopy (SEM)

The macroalgal surface and cross sections were prepared for SEM by fixation with 2% formaldehyde in seawater. After dehydration in an ascending ethanol series (30%, 50%, 70%, 90%, and 100%; v/v) they were critical point dried with carbon dioxide (Balzers CPD030) and sputter coated with gold–palladium (Balzers Union SCD004). Specimens were examined in triplicate with a scanning electron microscope (Zeiss DSM960), and pictures were taken with a Contax SLR camera.

### Molecular Analysis

Sections from diseased thalli were checked microscopically for the presence of typical phytomyxid structures like resting spores and plasmodia. Microscopically positive samples were transferred to sterile 1.5 mL tubes and DNA was extracted using a CTAB - Chloroform/Isoamylalcohol based DNA extraction protocol [35]. The primers used for PCR amplification were Mau2F (5′ ACGGGTACGAGGGACGTGGG) and Mau9R (TGCATCAGTGTAGCGCGCGT). These primers were designed using the primer blast tool (http://www.ncbi.nlm.nih.gov/tools/primer-blast/). The *Maullinia ectocarpii* sequence AF405547 was used as input sequence. The length of the product was specified between 500 and 1800 bp, and 20 primers which were checked against the nr database excluding environmental uncultured sequences were recovered. These primers were aligned to an extended phytomyxean alignment and the theoretically best primer pairs were selected. The here listed primers Mau2F and Mau9R allowed direct sequencing of the 18S rDNA of the here discussed *Maullinia* spp.

PCR-reactions (20 µL) were run in a reaction mixture containing final concentrations of 0.2 mM dNTPs, 0.1 µM of each primer, 1 × PCR-reaction-buffer without MgCl_2_, 2.5 mM MgCl_2_, 2 µg mL^−1^ BSA (Bovine Serum Albumin), and a final concentration of 0.5 U of Taq-Polymerase (Dream Taq, Fermentas 5 Units µL^−1^) and 3 µl DNA.

A touchdown PCR- protocol was used: 96°C for 4 min initial denaturation, followed by two cycles of 96°C for 25 s, 65°C for 25 s and 72°C for 1.5 min followed by two cycles each with a primer annealing temperature of 60°C and 58°C and finally 30 cycles with a primer annealing temperature of 54°C and a final slope of 72°C for 10 min. Samples were purified, and directly sequenced using the BigDye 3.1 chemistry according to the manufacturers instructions, and were analysed using the ABI 3130 Genetic Analyser. The 18S rRNA gene sequences obtained in this study were deposited in NCBI GenBank under the accession number JX163857.

Sequences were aligned using the ClustalW plugin for geneious (Biomatters Inc.) and the alignment was subsequently improved manually. For phylogenetic analyses we used the MrBayes plugin in geneious.

## Results

Abnormal growth and gall development on the surface of the thallus of *D. antarctica* was observed in wild populations at Coliumo bay at the central coast of Chile (Fig. 1, [36]) and compared to healthy individuals.

### Healthy Individuals

**Figure 2:**
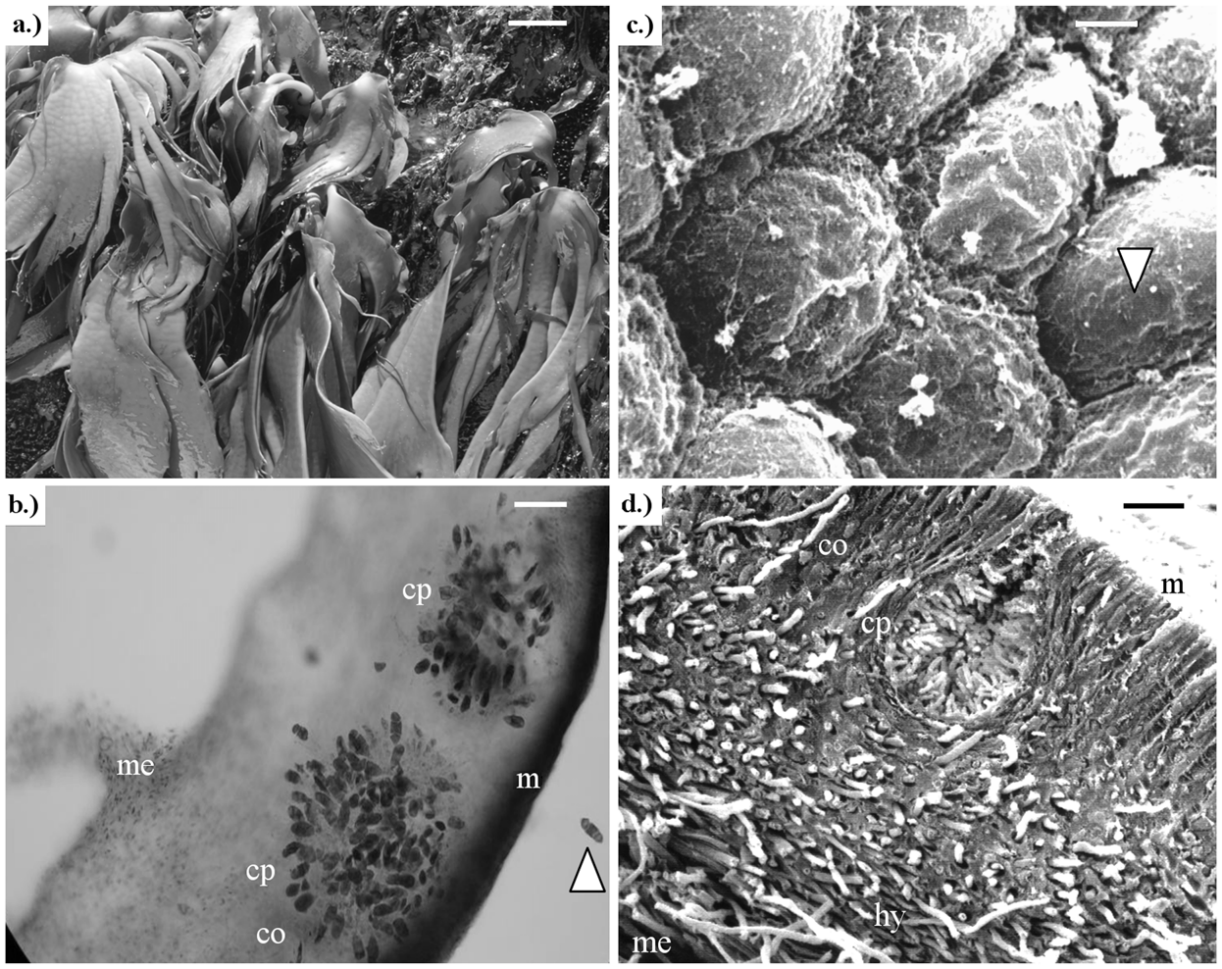
*Durvillaea antarctica* occurring at the central coast of Chile. **a)** Healthy population of *D. antarctica* in the natural environment; **b)** Light microscopic microphotograph of a cross-section of a dioecious frond of *D*. *antarctica* (stained with aniline blue) showing two female conceptacles (cp) with one free oogonium (white arrow), meristoderm (m), cortical (co) and medullary (me) zones in a normal frond; **c)** Scanning electron microphotograph (SEM) with details of the surface of the thallus, and cells disposition in the algal surface (arrow shows one cell); **d)** Detail of a cross-section of a normal thallus using SEM showing early stages in conceptacle development (cp), meristoderm (m), cortical (co) and medullar (me) tissue with normal swift hyphae (hy). Scale bar: a) 10 cm; b) 100 µm; c) 2 µm; and d) 50 µm.

Healthy fronds of the macroalgae were characterized by firm, elastic, smooth, and shiny thalli, in which the colour varied within the normal range of brown to dark-olive tones (Fig. 2a). In cross sections meristoderm, cortex and medulla was observed (Fig. 2b). As described by Naylor [31], the meristoderm consisted of a layer of 5–6 small, polyhedral, brick-shaped cells (Fig. 2c–d). The cortical zone was formed by regular radial rows of 8–15 elongate cells, and the medullary zone of irregularly interwoven hyphae (Fig. 2b, d), from which air-filled cavities separated by septa originated [3], [34]. Gametes in antheridia and oogonia (in a male or female fronds respectively), differentiated inside ovoid conceptacles that develop in the cortical zone with a small ostiole that opens to the surface (Fig. 2d) as described by Hoffmann and Santelices [3]. In transversal sections of a reproductive frond, one layer of conceptacles was observed (Fig. 2b, d).

### Diseased Individuals from Coliumo Bay

**Figure 3:**
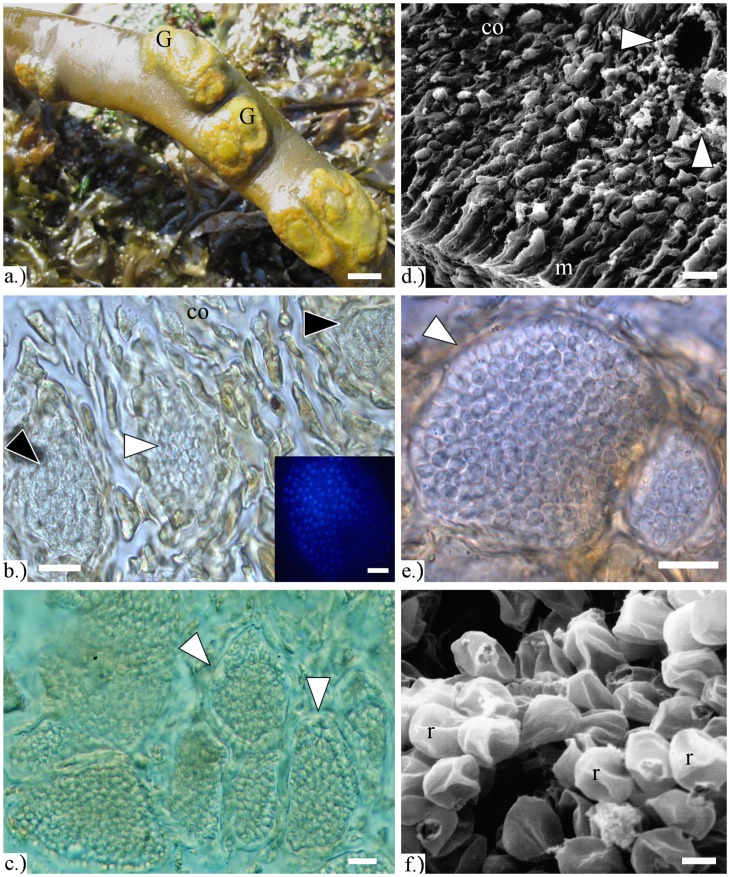
Infected *Durvillaea antarctica* samples from central Chile. **a)** Gall-like structures (G) on infected fronds. Bar 1 cm. **b)** Enlarged *D. antarctica* cells in the cortical zone (co) filled with resting spores (white arrow) and plasmodia (black arrows) of *Maullinia* sp. The two plasmodia are in the process of developing into resting spores, because the beginning formation of the cell walls of the cystosori can be seen. Inlay: DAPI staining of a multinucleate plasmodiums which is in the process of developing into resting spores. Bar 10 µm. **c)** Resting spores of *Maullinia* sp. (Light microscopic image, DIC). Masses of single resting spores are completely filling the enlarged cells of *D. antarctica*. Bar 10 µm. **d)** SEM-micrograph showing the formation of the cortex (co), meristoderm (m), and enlarged cells infected by *Maullinia* sp. (arrows). Bar 20 µm. **e)** Resting spores of *Maullinia* sp. (Light microscopy, DIC). The resting spores are smooth, thick walled, and roundish but slightly irregular in shape. Bar 10 µm. **f)** SEM microphotograph of the smooth walled resting spores of *Maullinia* sp. infecting *D. antarctica* samples from central Chile. Bar 2 µm.

Diseased fronds were recognized by the presence of yellowish protruded, wart-like structures of scattered tissue, irregular in form and size, however, tending to be circular or elliptic, with a diameter between 0.5–4 cm (Fig. 3a).

Structures formed by the phytomyxean parasites were observed in the yellowish brown subcortical tissue from the wart-like lesions formed on the fronds of the alga (Fig. 3b–f). Initially infected cells appear to be paler than healthy cells. Within these cells the plasmodia of the phytomyxean parasite develops (Fig. 3b). The plasmodia are colourless and vary in size and shape. Host cells infected by the parasite enlarge until they are about three to four times the size of an uninfected cell (Fig. 3c). Plasmodia lack obvious morphological characteristics until they start to develop into resting spores. However, the plasmodia are multinucleate and undergo synchronous cell divisions during the growth of the pathogen (Fig. 3e) until the plasmodium starts to develop into resting spores. In some cells this transition was clearly visible in light microscopy. The resting spores which develop from these plasmodia are smooth, thick walled, slightly irregular in size and shape and are not aggregated into cystosori (Fig. 3e, f). The resting spores are on average 3.3+/−0.3 µm across the broadest plane with a minimum of 2.8 µm and a maximum of 4.0 µm across the broadest plane (n = 30). Phytomyxea have a complex, multi stage life cycle of which we could observe the sporogenic plasmodia, plasmodia which were in the process of developing into resting spores, and enlarged host cells filled with resting spores, but we were not able to identify zoosporangia or primary plasmodia.

**Figure 4:**
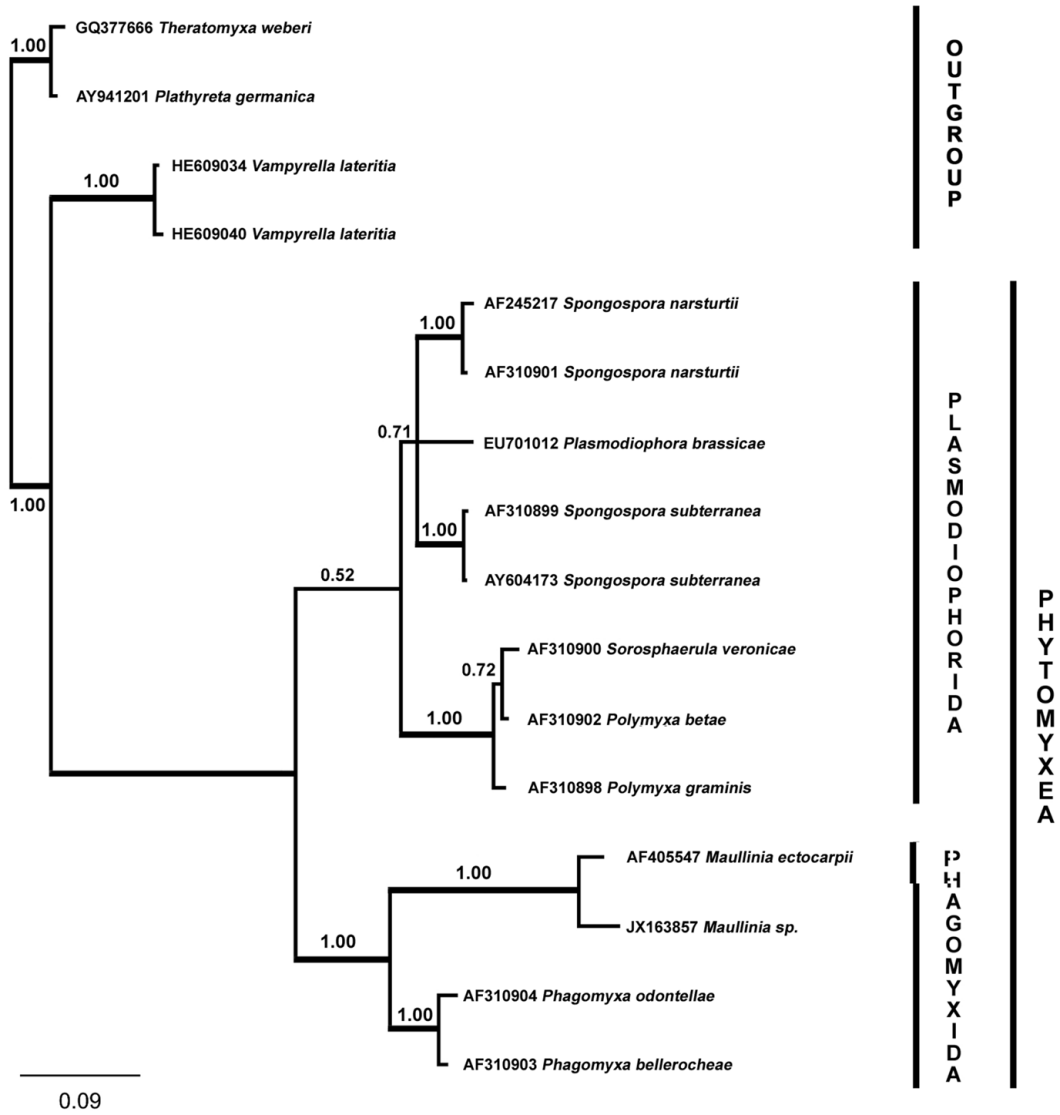
Phylogenetic tree of the partial 18S rDNA gene sequences from the novel *Maullinia* sp. which is parasitic in *Durvillaea antarctica*. *Maullinia* sp. (JX163857) is placed within the Phagomyxida and sister to the second brown algal parasitic species of Phytomyxea *Maullinia ectocarpii*. The phylogenetic tree was constructed using Bayesian analyses running the GTR+I+R model with 1000000 generations (burnin = 1000). Posterior probabilities are shown above the nodes. The resolution of the branches within the Plasmodiophorida was better supported in PHYML analyses (data not shown).

To demonstrate the affiliation to and the placement within the Phytomyxea a phylogenetic analysis was conducted. A phylogenetic tree was constructed using Bayesian analyses running the GTR+I+R model with 1000000 generations (burnin = 1000). In the resulting posterior output tree the new parasite is placed within the Phytomyxea and is sister to *Maullinia ectocarpii* (Fig. 4). However, hosts of *M. ectocarpii*, also brown algae, share the same habitat with *D. antarctica*. Therefore, whether or not the phytomyxean parasite described in this study is a new species or if the here described symptoms are an alternative life cycle of *M. ectocarpii* remains to be resolved by more targeted future research. To avoid the introduction of “phantom species” we suggest referring to the *Durvillaea antarctica*-phytomyxid as *Maullinia* sp. until a reliable species delimitation is possible.

## Discussion

Phytomyxea are a group of obligate biotrophic parasites. Although they were recognized as protists when the group was first established, they were considered to be basal fungi for a long time [37]. Therefore, these organisms were mostly referred to as “Plasmodiophorids” to avoid taxonomic placement, a name which is still commonly used for this group. However, recent studies based on both morphology and multi-gene phylogenies place the Phytomyxea in the Eukaryote Supergroup Rhizaria [37], [38]. Based on molecular data, Phytomyxea are divided into two groups: The Plasmodiophorida, consisting of parasites of green plants and Phagomyxida, parasites of brown algae and diatoms [39], [40]. This division was already established by Karling [41] however the distinguishing factor of the two groups used then was the presence and absence of a complete life cycle that terminates in the formation of the characteristic resting spores. Until now both molecular data and the absence of the formation of resting spores were coherent. However, the phytomyxean parasite described here forms resting spores and based on molecular data it clearly belongs to the Phagomyxida. This is the first solid report of the production of resting spores by a phagomyxean parasite. Therefore, the concept used by Karling [41] is not longer applicable as delimitating factor for the two phytomyxean orders as the data presented here show that at least some species within the Phagomyxida have a complete life cycle that terminates in the formation of resting spores.

Within the Phagomyxida so far 4 species have been described. *Phagomyxa bellerocheae* and *Phagomyxa odontellae* are parasites of diatoms [42]. In brown algae, *Phagomyxa algarum* was found as parasite of *Bachelotia antillarum* (formerly *Pylaiella fulvescens*) and *Hincksia mitchelliae* (as *Ectocarpus mitchelliae*) in North Carolina, USA [43]. *Maullinia ectocarpii* was identified from the thalli of *Ectocarpus siliculosus* collected from the south of Chile and has the ability to infect several brown algal hosts [28]. But resting spores were not described from any of these species. However, when the 18S rDNA sequence from the *Maullinia* sp. described in this study is compared to the 18S rDNA sequences from *P. bellerocheae*, *P. odontellae* and *M. ectocarpii* it becomes evident that the phytomyxid parasite from *D. antarctica* groups with *M. ectocarpii*. This is not surprising, as both are parasites of South-American brown algal hosts. The tree also indicates that the *Durvillaea*-parasite is likely to be a novel species, but for the description of the new species DNA data from more isolates along with ultrastructural analyses of the developing parasite will be needed.

The phylogenetic and microscopy results strongly support that also Phagomyxida have a complete life cycle that terminates with the formation of persistent resting spores. Therefore it might be possible that the so far described phagomyxid species were not yet assigned to their primary host. The current species concept in Plasmodiophorida defines the primary host as the plant taxon in which the full life cycle including the resting spores can be observed in contrast to “alternative hosts” in which only the primary plasmodia and zoosporangia are formed, consequently raising the question whether or not this concept is true for Phagomyxida and the primary host taxa for the other described species have yet to be discovered. Although Schnepf et al. [42] and Maier et al. [28] searched thoroughly for all stages of the phytomyxid life cycle; they were not able to find resting spores in any of their samples or hosts tested. The presence of resting spores in Phagomyxida adds a whole new dimension to the ecology of phytomyxids in marine environments especially their ecological impact within coastal kelp forests, because the here described resistant resting spores allow the parasites to survive adverse conditions for a long time, but also help them to spread by being carried with the currents over long distances [44].

Based on previous biomechanical studies, *Durvillaea* spp. are considered as one of the strongest algae in the world [45]. The special morphology, together with high level of alginate as important component in new tissue produced within the entire blade, allows *D. antarctica* to be elastic and flexible, necessary features to withstand the rigours of the extreme high hydrodynamic environment in which this macroalga lives [11]. Thus, diseases that result in galls, holes and hardness of the thallus (Fig. 3a) interact with these vital features and consequently can affect the survival of the host [27]. A number of recent reviews pointed out the importance to reassess the largely unexplored influences of algal parasites, suggesting that they occur undiscovered in natural populations of algae, but strongly impacting the ecosystems these algae create as a whole. These parasites are increasingly being considered to be equally important to predators for the functioning and the stability of these important coastal ecosystems [17], [20], [46]–[48]. The galls produced by the phytomyxean parasite described here clearly can impact the important bull-kelp belts directly by changing the stability of the bull kelp and indirectly by altering the ecosystem created by the bull-kelps.

We found individuals of the bull-kelp alga *Durvillaea antarctica* presenting galls in the same geographic region of the prior study of Aguilera et al. [27] twenty three years ago (Fig. 1). This suggests that this parasite might be present along the whole central coast of Chile and that this parasite is a persistent factor of these bull-kelp algal belts. More research on this pathogen, its life cycle, its abundance and distribution is needed to reliably estimate the positive and negative impacts that this parasite might have.

Although the cause for development of galls in *D. antarctica* has not been unequivocally established, and because Koch’s postulates were not yet fulfilled in the lab, the fact that no rhizoids, hyphae, exit papillae or exit tubes were visible, helps to differentiate the observed endophyte from other parasitic organisms belonging to the Chytridomycota [49] or Oomycetes [50] (Fig. 3a–f). Our observation strongly suggests that the phytomyxean parasites trigger the formation of the galls. Upon infection, many Phytomyxea - including the closely related *Maullinia ectocarpii* - cause enlargement of the cells they infect (for a comprehensive list of species causing hypertrophies see [51]). Multinucleate plasmodia were observed in enlarged host cells where they developed into resting spores (Fig. 3e, f). No phytomyxean structures could so far been found in other areas of the host than the galls, however more targeted studies will be needed to identify the full life cycle and host range of this novel *Maullinia* sp.

Microbial parasites of algae are typically characterized by their small size, short generation times, high rates of reproduction, and a simple life cycle often limited to a single host [48]. But Phytomyxea on the other hand are characterised by a complicated life cycle involving two free swimming zoosporic stages, two plasmodial stages that are linked to the host, a thin walled zoosporangial stage, and thick-walled resting spores [44]. Therefore, detection, isolation and cultivation of those pathogens are problematic if not impossible, especially when they infect large hosts like *D. antarctica* which cannot be cultured in a controlled laboratory environment [17], [50]. Until now, very little is known about the ecology of macroalgal pathogens [51]–[53], about the mechanisms of pathogenesis and disease resistance, or what the effects on early stages of *D. antarctica* are. Quantitative data about the numbers of diseased individuals in Chile and the global distribution of this *Maullinia* sp. are still missing. Knowledge about all these factors is needed to understand the impact of phytomyxean parasites on kelps in general. For the Laminariales heavy parasite attacks on the microscopic stages of host have been already proposed as being a regulatory factor for the population dynamics and the establishment of these important key species [54]. We have just begun to explore the vast biodiversity of pathogens in the sea, and the possible roles in marine ecosystems [44]. Recent environmental 18S rDNA surveys of microbial eukaryotes have unveiled a large reservoir of unexpected diversity in pelagic systems and emphasized their ecological potentials for ecosystem functioning [55]. Besides of the detrimental effect, zoospores from oomycetes, phytomyxea, chytrids and other parasites provide an understudied link between producers and secondary consumers and opened new perspectives in the context of food-web dynamics [48], [55], [56].

Combining microscopy, cultivation, infecting experiments and up-to date “omics” approaches, offers a huge potential to improve our knowledge about these parasites [17]. A highly targeted approach is needed in order to understand and describe this novel, large kelp infecting species. Our results show that in so important resources such as brown algae unrecognized and so far not described parasites exist.
